# Characterizing the Structural Behavior of FRP Profiles—FRCM Hybrid Superficial Elements: Experimental and Numerical Studies

**DOI:** 10.3390/polym14061076

**Published:** 2022-03-08

**Authors:** Amir Reza Eskenati, Amir Mahboob, Ernest Bernat-Maso, Lluís Gil

**Affiliations:** 1 Strength of Materials and Structural Engineering Department, Polytechnic University of Catalonia, C/Colom 11, TR45, 08222 Terrassa, Spain; amir.mahboob@upc.edu (A.M.); ernest.bernat@upc.edu (E.B.-M.); lluis.gil@upc.edu (L.G.); 2 Serra Húnter Programme, 08222 Terrassa, Spain

**Keywords:** FRCM, pultruded FRP profile, hybrid element, numerical simulation, experimental tests

## Abstract

Composite materials have been increasingly used to produce hybrid structures together with concrete. This system is commonly applied to bridges and roof structures. The main idea of the current research was to extend this approach by replacing the concrete with a fabric-reinforced cementitious matrix (FRCM) composite, resulting in a combination of composite materials. The main aim was to characterize the structural behavior of fiber-reinforced polymer (FRP) profiles and FRCM hybrid superficial elements. Two different prototypes of the hybrid superficial structural typology were tested to cover bidimensional and three-dimensional application cases of the proposed technology. After mortar cracking, the experimental results revealed a ductile response and a high mechanical capacity. A finite element model was implemented, calibrated, and validated by comparing numerical data with experimental results of the two prototypes. The output was a validated model that correctly captured the characteristic response of the proposed technology, which consisted of changing the structural response from a stiff plate configuration to a membrane type due to cracking of the FRCM composite part of the full solution. The suggested numerical model adequately reflected the experimental response and proved valuable for understanding and explaining the resistive processes established along this complicated FRP-FRCM hybrid structure.

## 1. Introduction

The most common composite materials for strengthening building structures are divided into two different types based on the matrix’s composition, organics (polymers) or inorganics (cement, lime). Among others, the usual types of composite material are fiber-reinforced polymer (FRP) and fabric-reinforced cementitious matrix (FRCM) for organic and inorganic types, respectively [1]. FRP materials provide many benefits over more conventional reinforcement methods, such as high strength-to-weight ratio, relative simplicity, fast installation, cost effectiveness, and high durability. Currently, FRP rods typically used in the aforementioned engineering are mostly carbon fiber-reinforced polymer (CFRP) and glass fiber-reinforced polymer (GFRP) fiber types. The cost of GFRP is relatively low. Long-term exposure in a complex service environment, on the other hand, will result in resin matrix deterioration, plasticization, and expansion, as well as fiber/resin interface debonding [2]. Relevant research has revealed that the ultimate elongation of C/GFRP increased significantly due to the uniform fiber hybrid dispersion, and C/GFRP represented a controlled and gradual failure compared to CFRP, indicating the obvious pseudo-ductility behavior, which was useful in providing a warning before the final failure of materials and structures [3].

FRCMs are sometimes favored over FRP systems depending on working conditions such as high-temperature or high-humidity environments, and these are preferred for masonry applications because of their mechanical, chemical, and physical compatibility [4,5]. FRCMs are made up of high-strength-fiber open-mesh textiles that are incorporated in inorganic matrices. Carbon, alkali-resistant (AR) glass, polyparaphenylene benzobisoxazole (PBO), or basalt fibers can be used to make the textile [6]. Tensile testing was carried out using the clevis grip method by varying the bonded length of the metallic tabs used to hold the ends of the specimens. They came to the conclusion that a bonded length of 150 mm was enough for characterizing FRCM composites. In addition, the performance and failure modes of FRCMs reinforced with numerous carbon fabric plies were studied [7].

FRP profiles have been used in recent years to produce new structures. FRP profiles are manufactured with a pultrusion technique [8]. This product has a wide range of applications in construction and building systems. Zou et al. [9] reported the application of pultruded glass fiber-reinforced polymer (GFRP) profiles in the FRP-concrete hybrid section of a bridge system. Using bolted connections and/or adhesive bonding had a positive effect on attaining the full composite section response. In another study, Zou et al. [10] investigated the behavior of bolted shear connections placed between FRP girders and ultra-high-performance concrete slabs under push-out tests. Two failure modes were observed: bolt shank shear and FRP flange shear-out. Nguyen et al. [11] investigated the effect of adding epoxy to bolted connections in FRP-concrete hybrid slabs and they found that the combination of epoxy and steel bolts led to an improvement in ultimate load bearing capacity.

Correia et al. [12] used epoxy in interfacial connections in GFRP-concrete hybrid beams. Experimental observations showed that the full exploitation capacity of the hybrid beam was limited due to concrete–adhesive interface failure. Koaik et al. [13] compared the effects of epoxy adhesive and bolt connection in reinforced concrete-GFRP hybrid beams experimentally. The combination of these connections prevented the shear failure at the interface. Moreover, the fatigue behavior of pultruded FRP profiles with bolted joints and epoxy resin joints was studied by Wingerde et al. [14]. A novel approach to dealing with FRP-concrete connections has been recently proposed by Mahboob et al. [15,16], who used epoxy resin to connect a flexible fiber fabric to carbon FRP parts and left the free parts of this fabric embedded in concrete, resulting in a ductile and superficially distributed FRP-concrete connection. The fabric used was analogous to the one used in FRCM systems.

In terms of FRCM components, several studies have been performed to investigate the performance of this composite material and its application as a strengthening system for masonry and concrete structures combining different types of fibers and mortars. However, the most promising alternative for the construction industry is the one including glass fiber because of its lower cost. Studies about the sufficiency of the glass fiber grid in different structures and infrastructures have been presented by Khodaii et al. [17], Meng and Khayat [18], and Falliano et al. [19]. Corradi et al. [20] investigated the connection between glass fiber grids and cement mortar in the shear strengthening of wall panels. Reinforced panels showed that the combination of glass fiber grids and mortar enhanced the mechanical response. Another study performed by Dalalbashi et al. [21] was aimed at evaluating the bond behavior of glass fiber grids-mortar experimentally and analytically.

In conclusion, hybrid structures using pultruded FRP profiles and concrete have already been studied and have started to be applied as commercial solutions. On the other hand, the FRCM system is widely used as a strengthening solution. In addition, the FRP-concrete connection method proposed by Mahboob et al. [15,22] opened the door to future FRP-FRCM hybrid structure technology. However, as per the authors’ knowledge, there are no published references that suggest combining pultruded FRP profiles and FRCM to produce structural superficial elements. The possible range of application of these hybrid structures may include urban elements, tunnel sustainment, or thin roofing systems. This new proposal aims to combine the structural performance and stiffness of FRP profiles with the shape adaptability and the ductile response associated with the cracking process of the mortar matrix of FRCM, which also shows great post-cracking strength. In this sense, proving the connection performance between these two composite components and the equivalent system structural system is mandatory.

Thus, the main objective of the current work was to describe and characterize the structural behavior of this new structural system composed of FRP profiles and FRCM. Experimental tests and numerical simulations were used with this aim.

## 2. Experimental Program

### 2.1. Specimen Description

**Figure 1:**
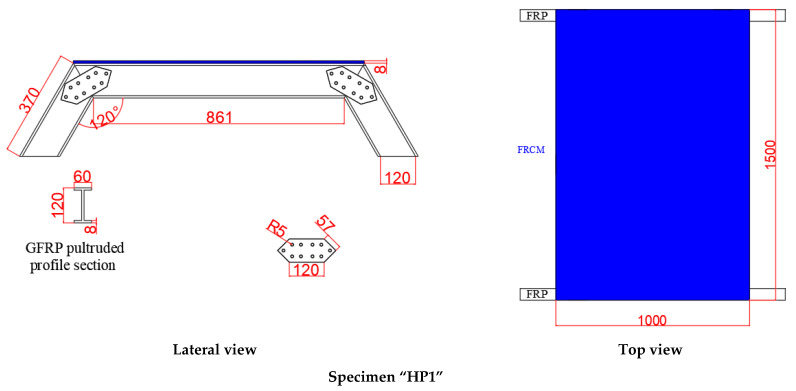

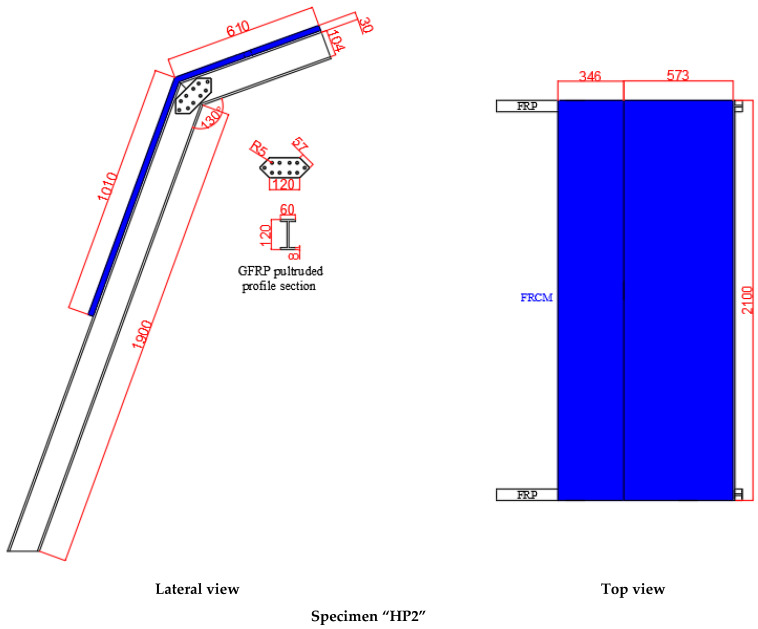
The geometry of the specimens (dimensions in mm).

The experimental program consisted of two hybrid panels with different geometry (see Figure 1). Both panels were made of IPE 120 GFRP profiles and FRCM, which was composed by fiber glass mesh and mortar. Steel bolts were used to connect GFRP profiles among them. The angles between the GFRP profiles (120° and 130°) were chosen to fall within a range of 120–160°, which was chosen to cover possible arched sustainment solutions of real tunneling sections. This angle was obtained by approximating a semi-circular arch with a finite (between 4 and 9) number of straight lines.

### 2.2. Materials

#### 2.2.1. Pultruded GFRP Profiles

Pultruded GFRP profiles (3 m long) were produced by Composites ATE S.L. (Barcelona, Spain). The profiles had an IPE120 cross-sectional shape and were made with a polymeric matrix of unsaturated polyester and thermosetting, which was reinforced with glass fibers of the E-glass type with a volume fraction of 68% [23]. These profiles were formed through a pultrusion process. The mechanical properties of GFRP-pultruded profiles were previously determined and these are summarized in Table 1. Note that the mechanical performance of these GFRP profiles might be lower than that of general GFRP because they were selected with the aim of promoting mechanical compatibility with FRCM, which is more flexible.

Connection plates used to join profiles in bolted joints were extracted from the web of the same profiles, so these elements had the same properties summarized in Table 1.

#### 2.2.2. Fiber Glass Mesh

The grid mesh formed by fiberglass A.R. (alkali resistant) disposed in square pattern (dimension of 25 × 25 mm^2^) was produced by Mapei S.r.l. Company (Milano, Italy). This fiber contained 17% zirconium oxide and was pre-primed. These two particularities improved the tensile strength, the durability, and the bonding with mortar/concrete matrix. Table 2 presents the mechanical properties of the GFRP grid experimentally obtained in [24].

#### 2.2.3. Mortar

The used mortar was a monocomponent Portland cement-based mortar reinforced with fibers and silica fume, which complies with the requirements of Class R4 of BS EN 1504-3 [25]. The commercial name is Sika^®^ MonoTop-612. This product is a low-permeability repair micro-concrete. The ingredients of this mortar include Portland cement, polymer redispersal powder, selected aggregates, and additives. Table 3 shows the available information about the mechanical properties of this mortar provided by the manufacturer and obtained from previous experimental tests.

#### 2.2.4. Screws, Nuts and Washers

M10 × 35 hexagonal head screws of quality Q12.9, M10 nuts, and M10 washers were used to fix the joints between the GFRP pultruded profiles.

#### 2.2.5. Adhesive

A thixotropic bi-component structural epoxy adhesive able to cure under indoor environmental conditions (Loctite EA 3425) was used to fix the FRCM mesh to the flanges of the GFRP profiles. This resin has a shear strength of 27 N/mm^2^ and a temperature resistance of up to 120 °C, as well as a considerable gap filling capacity of up to 3 mm.

### 2.3. Manufacturing Process

The manufacturing process consisted of the following steps:

(a) Cutting GFRP profiles to the desired length and angle. Drilling the holes for the connection. Repeating the process for the connection plates.
(b) Connecting GFRP profiles with a bolted connection including a connection plate on both sides of the web. Fixing GFRP profiles to the external reaction frame.
(c) Bonding the fiber fabric onto the top part of the top flange of the corresponding GFRP profiles to the desired length.
(d) Installing a wooden formwork (HP1) or a foam back surface (HP2) to support the production of the FRCM plate.
(e) Casting (HP1) or spraying (HP2) mortar over the fiber grid to produce the FRCM plates.
(f) Curing mortar for 28 days with daily superficial water spraying during the first week.

### 2.4. Testing Procedure

The structural characterization tests consisted of applying an imposed displacement and registering the reaction force and the structure deformation at 50 Hz for both specimens.

**Figure 2:**
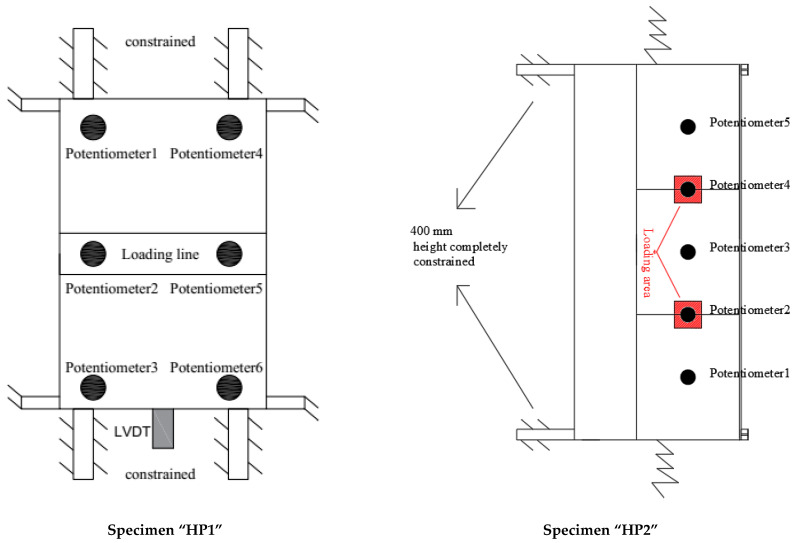
Setup of the monitoring and loading test for the hybrid structures. Potentiometers indicated as P and LVDT indicated as H.

**Figure 3:**
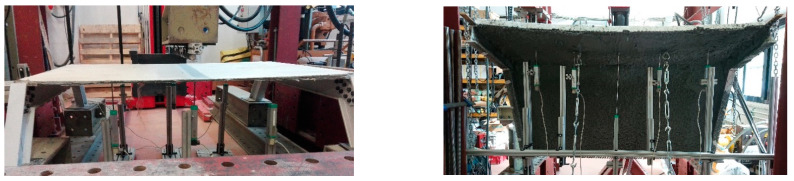
Specimens HP1 (**left**) and HP2 (**right**) before testing.

Specimen HP1 was simply supported on thin rubber pads that restrained horizontal displacements of the supporting ends. In addition, it was longitudinally restrained by fixing the horizontal GFRP profiles to the external load frame (see Figure 2). For testing specimen HP1, a hydraulic actuator (200 kN force range) was used to apply the displacement at the midspan of the FRCM plate at a rate of 1 mm/min. This action was distributed along the width of the specimen with a loading beam (HEM 200) that had an aluminum profile and a bar under it so as to concentrate the applied load in a line that was precisely placed at the desired position (see Figure 3).

The displacement of the actuator was measured with an internal LVDT of 250 mm range and 0.2% linearity. In addition, there were 7 sensors that were used to monitor the test in specimen “HP1.” In addition, 6 potentiometer sensors (10 cm range and 0.02% accuracy) were spread under the FRCM plate: 3 sensors were placed along each side edge at 8 cm apart from the edge (see Figure 2) and separated 25 cm among them in the longitudinal direction with the central one centered under the displacement imposition line. In the longitudinal direction, one LVDT with a 20 mm range and 0.02% accuracy was placed in contact with one of the horizontal GFRP profiles, at its central position, to control the effectiveness of the longitudinal fixation of the specimen to the loading frame.

Specimen HP2 was completely fixed at the lower part of the vertical-like GFRP profiles by bolting them to the external loading frame. In addition, the horizontal-like GFRP profiles were horizontally and vertically restrained with steel chains (see the sketch in Figure 2). The load was applied indirectly as an imposed displacement at two 150 mm square areas located three-thirds of the way down the length and transversal midspan of the top FRCM plate. This displacement was manually applied through tensioners that act on steel plates placed at the extrados of the structure. These tensioners were fixed to a reaction beam of the rigid loading frame through hinges. The direction of the applied displacement/load was orthogonal to the top FRCM plate. The applied load at both areas was simultaneously measured (load cells in the 10 kN range), recorded, and manually balanced by alternating the tensioners to be activated. Five potentiometers (10 cm range and 0.02% accuracy) were placed in line at a sixth of the length of the specimen, all at the same distance from each other. The 2nd and 4th potentiometers were placed as close as possible to the load application position. All potentiometers measured the vertical displacement.

## 3. Finite Element Modeling

**Figure 4:**
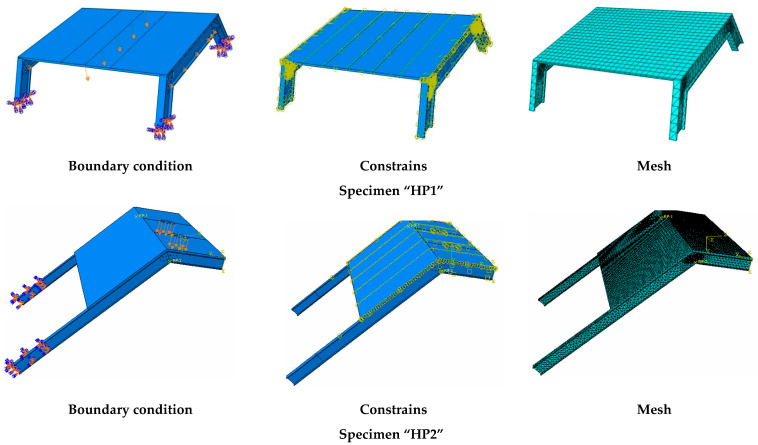
Overview of the numerical models.

The finite element model (FE) was implemented with commercial simulation software (ABAQUS^®^ 2020) so as to be easily exportable to any other simulation tool. Simulation of HP1 was used to calibrate the model, whereas simulation of the more complex HP2 case was used to validate the model. Figure 4 shows the outline of the FE (representative) models including boundary conditions, connections, and element mesh.

The various parts were modeled according to the geometric characteristics of experimental specimens described in the previous section (see Figure 4). Simplified cylindrical parts were set to model bolts in the HP1 case. Nuts and washers were not represented. According to the experimental and numerical results of the HP1 case (see Section 4 and Section 5), it was decided not to model the bolted connections of the HP2 case and replace them with a tie contact between profiles. Nevertheless, a further study would be necessary to completely characterize the FRP-FRP bolted connection in terms of possible local effects not considered in this research.

Bolts of the HP1 case, GFRP connection plates of the HP1 case, GFRP profiles, and mortar were modeled with linear hexahedrons with full integration of C3D8R elements. The truss element (T3D2) of a circular 1.05 mm^2^ section was considered for modeling the lines representing the glass fiber grid in the principal loading direction. Orthogonal wires were not considered, to simplify the model, although it may be interesting to include them in future research in which a three-dimensional response would be more relevant. In order to decrease the calculation time below 24 h, the size of the mesh for all parts was set to 110 mm. Implicit static analysis was implemented.

Regarding the contacts, the tangential behavior between bolts, connection plates, and GFRP profiles in the HP1 case was modeled as a surface-to-surface contact that used friction formulation (friction coefficient = 0.2, penalty approach). The interaction between the mortar and glass fiber grid was defined as an embedded region (total compatibility of strains), and the connection between the FRCM plate and GFRP profiles was modeled with a tie constrain (total compatibility of strains).

Load was modeled as an imposed vertical descending displacement in a line at midspan for the HP1 case and as an imposed displacement orthogonal to the top FRCM plate surface in the corresponding loading area (2 square areas of 15 cm edge) for the HP2 case.

The bottom supports of the GFRP profiles were considered to be fixed in both simulations. This approach completely represented the real behavior of the HP2 case, although it restrained the possibility of the support rotation in the HP1 case that was never observed during the experimental test. Finally, elastic supports (vertical and horizontal) of the top profiles of specimen HP2 (see Figure 4) were also considered in the simulation as an elastic foundation simplification with a stiffness of 0.01 N/mm and 0.001 N/mm for the vertical and horizontal respectively. The representation of the loads and the boundary conditions can be observed in Figure 4.

Regarding the mechanical properties of the implemented materials, GFRP was defined as an elastic homogeneous material characterized by its nine engineering constants (summarized in Table 4). The steel of the bolts (HP1 case) was modeled as a homogeneous elastic-plastic material. The parameters defining the plastic behavior of bolts are gathered in Table 5. Additionally, the stress–strain curve of the damage plasticity, compression damage, and tension damage were assumed for mortar material. Table 6 presents the parameters of mortar damage plasticity. The Young’s modulus and Poisson’s rate of mortar were considered 10 GPa and 0.2, respectively. Using a lower value of Young’s modulus than the provider’s data (see Table 3) is justified because the mortar bore tensile stresses in most of the test instead of compressive stresses was used to quantify this magnitude by the provider. Finally, the glass fiber of the grid was defined as an elastic-perfect plastic material with a Young’s modulus and Poisson’s of 61,250 MPa and 0.2, respectively. This fitted value of the Young’s modulus is in the range of the experimentally determined one (see Table 2). The yield stress was set to 676.8 MPa, which is the experimental value of the direct tensile test (Table 2). Finally, a softening response of the mesh after yielding (500 MPa of stress for a plastic strain of 0.1%) was defined to represent progressive tensile breaking of the mesh tows. All these values are in the range of the ones indicated by manufacturers (see Section 2) or experimentally determined and were fitted to calibrate the HP1 case.

## 4. Experimental Results and Discussion

First, it has to be noticed that the results presented herein and the discussion based on them might be affected by variability, and it is suggested to take into account that only one specimen per type was tested when analyzing the provided data. Further future experiments might be required to confirm these results.

**Figure 5:**
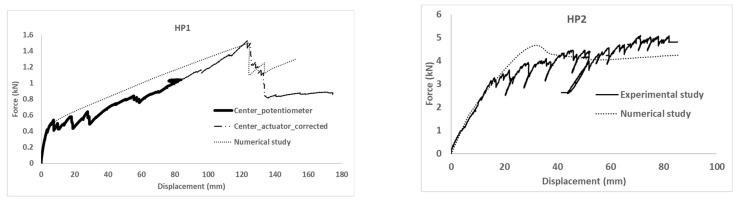
Force vs. displacement plots for experimental specimens and their numerical verification.

Experimental results in terms of force–displacement curves are presented in Figure 5. The experimental response of the HP1 test is plotted in two parts. The first one (solid) shows the recorded force versus the average displacement of the potentiometer sensors placed below the loading line. Connected to this first part, the second part (thicker dotted line) represents the applied force versus the displacement of the actuator after correcting this last one according to the difference between the potentiometers during the first part of the test. This change is required because the deformation of the specimen was over the measuring range of the installed potentiometers.

**Figure 6:**
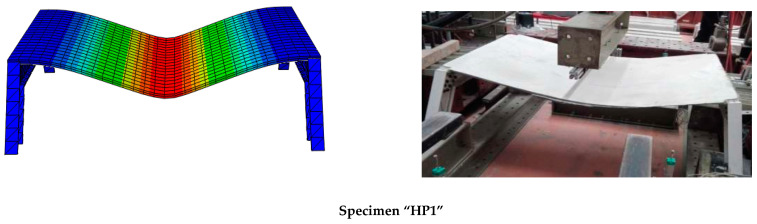

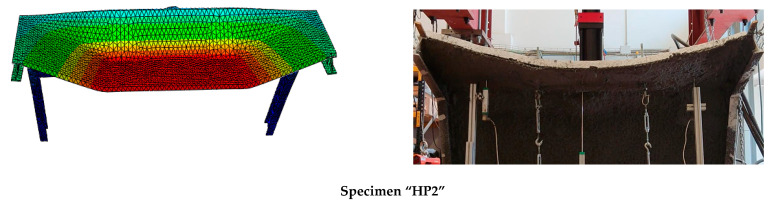
Failure mode in the tests.

The HP1 response was characterized by a first initial lineal response in which the observed deformation of the specimen corresponded to two opposite edges of the fixed plate. This stiffer behavior was maintained up to the crack of the mortar along the two edges defined by the FRP-FRCM connection. From this point and on, the observed deformation corresponded to the typical parabolic description of a plate that is simply supported at two edges. Progressive FRCM cracking, which is observed with the upward and downward trends in the experimental curve in Figure 5, changed the mechanical qualitative response from plate-like to membrane-like, in which the mortar contribution could be ignored and the wires of the glass fiber grid bore the load until the final failure, when some of the wires broke in tension, causing the last sudden load decrease (see Figure 6). Thus, in the HP1 case, the failure mechanism was reaching the tensile strength of the fiber mesh after the FRCM was completely cracked and it was acting similar to a membrane. In conclusion, the tensile failure of FRCM defines the global failure of the HP1 specimen. 

The ductility of the HP1 specimen was qualitatively evaluated by comparing the displacement at the maximum load (122 mm) with the displacement when the cracking process started (8 mm). The former one was 15 times greater than the second one.

Hence, the FRP-FRCM connection manufactured by the adhesive bonding of the FRCM mesh to the FRP profile before mortar casting was effective enough to support the loading process up to the FRCM tensile failure. In addition, no significant deformations or damage were observed in the FRP-FRP bolted connection of the HP1 specimen. Hence, it is concluded that the part that had the most influence on the mechanical response of the tested hybrid FRP-FRCM specimen was the FRCM plate. This observation proved the effectiveness of the FRP-FRP and FRP-FRCM connections proposed in this novel technology and turned the analysis far from the FRP-concrete connection typically analyzed for current hybrid structures (see, for example, the large plastic bolt deformations found in the load–slip curves by Di et al. [27] and Rajchel et al. [28] or the load–slip curves of FRP form-concrete elements by Gong et al. [29]). Finally, comparing the experimental observations of HP1 tests with the idealization of the shear behavior of the FRP-flexible mesh-concrete connection presented in [16], which is the closest case to the novel tested technology, it is concluded that the FRP-FRCM connection worked in the elastic range during all tests of the HP1 specimen.

The HP2 mechanical response is also plotted in Figure 5. This plot represents the vertical displacement of the displacement sensors placed closest to the loading areas (average value of Potentiometer 2 and Potentiometer 4) versus the addition of the load recorded by the individual load cell installed in each tensioner. The test of the HP2 specimen was finished when the maximum deformation of the tensioners (over 80 mm) was reached. HP2 behavior included a first lineal branch followed by multiple cracking formations. This progressive failure process can be identified by the upward and downward trends of the experimental load–displacement curve. This fact is supported by the visual and acoustic observations made during the test. After reaching the maximum load (around 5 kN for a vertical deformation of 70 mm), the cracking process continued and the load was kept almost stable with a slight decrease. Specimen HP2 did not reach the failure stage as long as the fiber mesh and GFRP profiles resisted until the end of the test, which was determined by exceeding the loading system’s maximum displacement capability. As a result, it was expected that the failure mechanism would surpass the FRCM’s tensile strength. The high ductility of this hybrid system, however, prevented it from reaching an ultimate failure state.

The ductility of the HP1 specimen was qualitatively evaluated by comparing the displacement at the maximum load (70 mm) with the displacement when the cracking process started (17 mm). The former one was four times greater than the second one. Increasing the thickness of the mortar matrix of FRCM plate had a significant impact on the load-bearing capacity of FRP-FRCM hybrid elements. Thus, the greater thickness of the mortar layer of the HP2 sample contributed to increasing the load-bearing capacity compared with the HP1 sample.

Comparing HP1 and HP2 specimens, it is clear that membrane formation previously observed in HP1 was not reached in the HP2 case. Thus, an idealization of the HP2 response should consider the FRCM part as a flexible plate that did not reach to develop the membrane response, because of insufficient damage to the mortar matrix. In fact, the observed cracks in the HP2 case were located around the loading areas, but these did not extend up to the FRCM-FRP connection edges (see Figure 6), thus retaining the plate-like structural response.

Again, as in the HP1 case, no damage or large deformation was observed in the FRP-FRP connections in the HP2 case. Moreover, the fiber grid was kept completely bonded to the FRP profiles during all the HP2 tests. Thus, it is also concluded in this case that the component part that most significantly controlled the mechanical response of this hybrid panel was the FRCM.

To sum up the experimental results in terms of influential parameters, it can be stated that the thickness of the mortar, the strength of the fibers, and the stiffness of the fibers are the main variables that may affect the structural response of hybrid FRP-FRCM systems. In particular, increasing the mortar thickness or increasing the stiffness of the fibers would increase the stiffness of the overall system and would delay the cracking stage. Moreover, increasing the strength of the fibers would only improve the ultimate load-bearing capacity, extending the ductility stage.

## 5. Numerical Results and Discussion

The force–displacement curves obtained from the numerical simulations are included in Figure 5, where the overlapping with experimental results can be observed. For both specimens, HP1 and HP2, the proposed numerical model successfully predicted the mechanical response of the experimental tests in terms of initial stiffness and maximum load.

**Figure 7:**
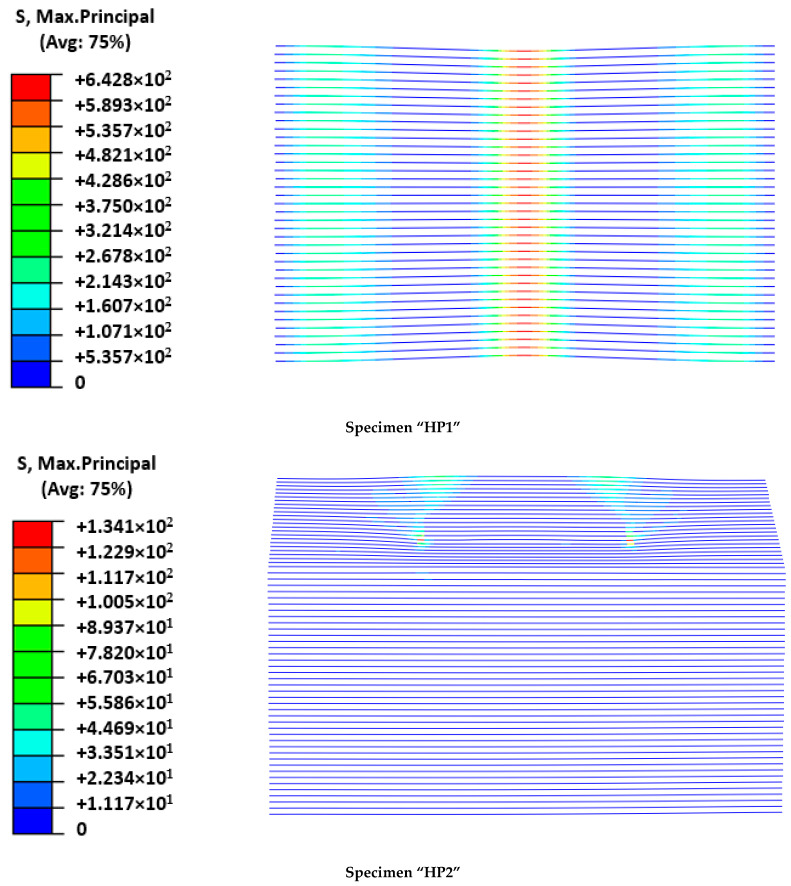
Stress distribution in the wire in the maximum load.

**Figure 8:**
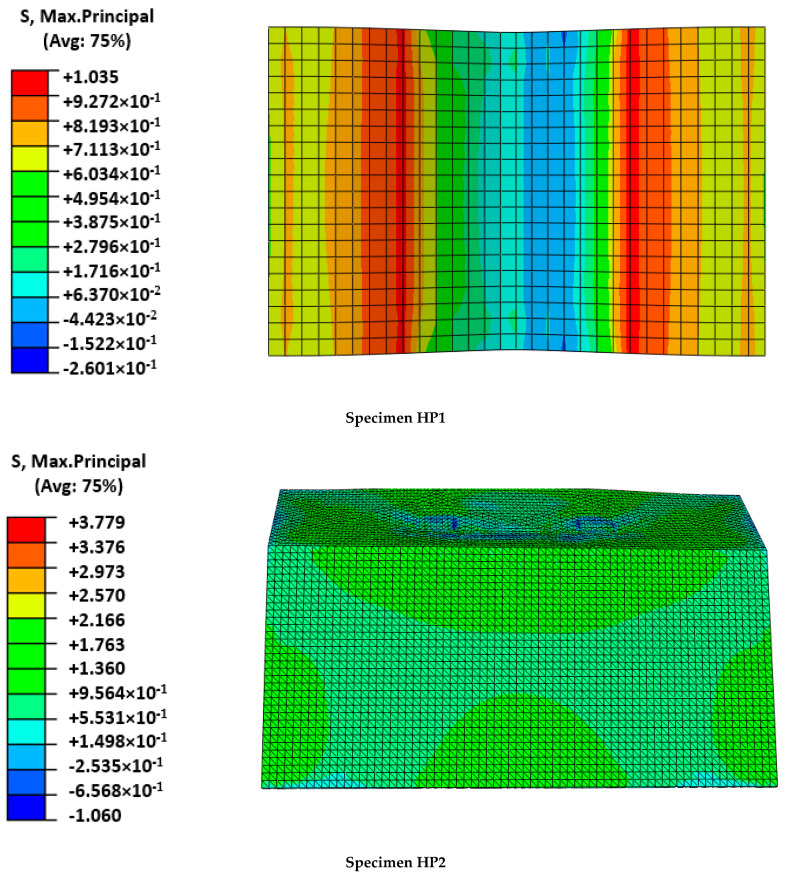
Stress distribution in mortar.

As expected, the fitting of the calibration case (HP1) was better than the fitting of the validation case (HP2). For the former, initial stiffness, post-cracking stiffness, maximum load, and load sudden drop were accurately predicted. In contrast, the predicted cracking load of the HP2 case was slightly overestimated and the post-cracking response was more conservative than the experimental output. Progressive cracking was not observed in simulations but was clearly identified in experimental results. This fact is justified by the experimental imperfections that contribute to a progressive asymmetric cracking of the mortar and the possibility of partial failure of the mesh. This is the reason that justifies the difference between numerical and experimental results in the HP2 of Figure 5. In contrast, the perfectly symmetric definition of the numerical models caused a slight increase in the cracking load that required the local mortar failure at several areas, simultaneously increasing the required external energy. Analyzing the failure modes (Figure 6) and the output results in terms of maximum principal stresses in the mesh (Figure 7) and mortar (Figure 8), a clear difference between the two cases arose: the HP1 structure reached the complete cracking of the mortar under the load application edge, whereas it was in progress at the end of the test of the HP2 case.

Observing the experimental failure mode of the HP1 specimen (Figure 6), it was clear that the FRCM behaved as a membrane in the last part of the experiment and the FRP-FRCM connections acted as hinges. The membrane response was also modeled by the simulation through a more concentrated deformation describing a sharper shape than the typical parabolic flexural one expected for plates. Regarding the calculated maximum principal stresses, it is clear that for the maximum load, the mortar around the loading edge in HP1 had failed (see Figure 8); maximum stress values were not under the load application edge but displaced on both sides, indicating that the mortar in the central area had broken because of reaching its tensile strength. It was experimentally observed that several cracks formed under the FRCM plate during the last loading stage, indicating the tensile failure of the mortar, as predicted by the numerical model. At the maximum loading instant, the mesh bore the load as a membrane and the mortar just covered it, so reaching lower stresses. In contrast, the stresses at the mesh (Figure 7) were concentrated at the midspan position reaching values of over 640 MPa, which were close to the maximum strength value defined in Table 2, indicating its tensile failure, as experimentally observed and recorded by the force sensors as a load drop (Figure 5).

Analyzing the HP2 case, it was observed that the model reproduced the deformation at maximum load, although experimental observations indicated a more evident plate-like response between the load application areas describing a parabola, whereas the numerical model predicted a flatter shape (Figure 6). This difference may indicate that the predicted mortar cracking was greater than the experimental observations. This would be a conservative approach. In fact, the stress level at the mortar (see Figure 8) overcame the mortar tensile strength and approached the flexural strength without reaching it. These outputs indicated that the flexural failure possibility, which was not experimentally observed, because the developed cracks had no continuity, was not reached even in the numerical model. Finally, the hypothesis that the HP2 case did not reach the point at which FRCM moves from behaving as a plate to behaving as a membrane was confirmed because the stresses in the mesh were far lower than its capacity (134 MPa vs. 676.8 MPa in Table 2).

**Figure 9:**
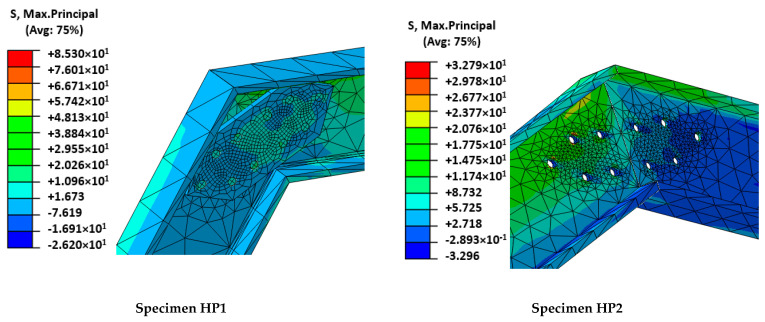
Stress distribution around connection area between FRP and FRP.

To continue with the analysis of the numerical results, it is observed that the maximum principal stresses in GFRP-pultruded profiles and in the FRP-FRP connection areas did not reach significant values (85 MPa and 33 MPa for HP1 and HP2 cases, respectively; see Figure 9) in comparison with GFRP mechanical capacities (Table 1). These results perfectly match experimental evidence that showed no plastic deformation, cracking, or any damage in GFRP profiles.

To sum up, it is concluded that the proposed model properly represented the structural response of hybrid FRP-FRCM structures, including mortar cracking and the possible change in structural response of the FRCM from plate to membrane. It also correctly captured the possibility of the tensile failure of the mesh and the mechanical contribution of the GFRP substructure through the accurate prediction of the load–displacement curves. Not considering the orthogonal mesh wires in the HP2 case, where a slight bidimensional response was observed, may partially explain why results as good as those with the calibration HP1 case, in which clear unidirectional response was registered, were not obtained.

Comparing the structural response of the two analyzed cases and considering that the free span of the HP2 case was greater than the span of the HP1 case, it was the HP1 case that resulted in more damage, even turning the response from plate to membrane. It is believed that two factors influenced this difference: first, the thicker mortar layer of the HP2 case (30 mm against 10 mm of the HP1 case) that contributed to extend the flexural load-bearing capacity of the mortar matrix of FRCM; second, the three-dimensional shape of the HP2 specimen contributed to a stiffer response of the FRCM plate, enhancing its performance as expected.

## 6. Conclusions

Experimental research and the corresponding numerical analysis for two tested specimens of FRP-FRCM hybrid superficial elements were carried out. Failure mode and structural-resistant configurations were analyzed by combining both experimental and numerical data. The experimental evidence matched the results of the implemented numerical model. Finally, the following observations may be made:

- Hybrid FRP-FRCM structures behaved linearly up to initial mortar cracking. After that, FRCM behaved as a plate with no linear response up to the flexural mortar failure. From this point and on, the FRCM behaved as a membrane whose main resistance mechanism was the tensile response of mesh tows up to their strength. A ductile response and high mechanical capacity after mortar cracking were observed.
- The three-dimensional definition of FRP-FRCM hybrid elements contributed to increasing the load-bearing capacity of the element. In the same line, increasing the thickness of the mortar matrix of FRCM plate had a significant impact on the load-bearing capacity of FRP-FRCM hybrid elements.
- The proposed numerical model accurately represented the experimental response. The predicted failure modes were consistent with those experimentally observed in terms of the structural mechanisms involved in resisting the load at the comparison point.

Finally, the novel proposed FRP-FRCM structural system proved to be able to resist large deformation states by maintaining and even increasing the load-bearing capacity through mobilizing different resisting mechanisms including nonlinear bending of the FRCM plate with progressive cracking or the residual membrane response defined by mesh capacity.

## Figures and Tables

**Table 1:** The mechanical properties of the GFRP profile [23].

Property	Value	Units	Testing Method
**Flexural**			
Ultimate strain	2.10 ± 0.05	%	EN ISO 14125:1998
Strength	734 ± 39	MPa	
Modulus of elasticity	35.0 ± 2.1	GPa	
**Tensile**			
Ultimate strain	1.37 ± 0.11	%	EN ISO 527-1:2012 EN ISO 527-4:1997
Strength	520 ± 27	MPa	
Poisson’s ratio	0.27 ± 0.02		
Modulus of elasticity	38.0 ± 1.4	GPa	
Effective shear modulus	3.98 ± 0.26	GPa	

**Table 2:** The mechanical properties of the glass fiber mesh experimentally obtained in [24].

Property	Value	Units
Tensile strength	676.8	MPa
Modulus of elasticity	60.95	GPa
Equivalent thickness of dry fabric ^1^	0.035	mm
Elongation at break	1.22	%

^1^ From manufacturer’s datasheet.

**Table 3:** The mechanical properties of Sika^®^ MonoTop-612 mortar.

Property	Value	Units	Testing Method
Density	2100	kg/m^3^	-
Tensile strength ^1^	2.90	MPa	EN1015-11
Flexural strength ^1^	6.56	MPa	EN1015-11
Modulus of Elasticity in Compression	23	GPa	EN13412
Compressive Strength ^1^	39.25	MPa	EN1015-11

^1^ From experimental tests published in [24].

**Table 4:** The mechanical properties of GFRP profile [23].

*E*_1_ (GPa)	*ν*_12_ = *ν*_13_	*ν*_23_	*G*_12_ = *G*_13_ = *G*_23_ (GPa)
10.77	0.27	0.33	3.98

**Table 5:** The mechanical properties of bolts.

**Characteristics**	Young’s Modulus (GPa)	Poisson’s Rate	Yield Stress (MPa)	Plastic Strain
**Value**	210	0.3	1080	0

**Table 6:** The parameters of mortar-damaged plasticity [26].

**Parameters**	Dilation Angle (°)	Eccentricity	f_b0_/f_c0_	K	Viscosity Parameter
**Value**	31	0.1	1.16	0.67	5 × 10^−5^

## Data Availability

The data presented in this study are available on request from the corresponding author.

## References

[1] Estevan L., Baeza F.J., Bru D., Ivorra S. (2020). Stone masonry confinement with FRP and FRCM composites. Constr. Build. Mater..

[2] Xian G., Guo R., Li C. (2022). Combined effects of sustained bending loading, water immersion and fiber hybrid mode on the mechanical properties of carbon/glass fiber reinforced polymer composite. Compos. Struct..

[3] Guo R., Xian G., Li F., Li C., Hong B. (2022). Hygrothermal resistance of pultruded carbon, glass and carbon/glass hybrid fiber reinforced epoxy composites. Constr. Build. Mater..

[4] Donnini J., Spagnuolo S., Corinaldesi V. (2019). A comparison between the use of FRP, FRCM and HPM for concrete confinement. Compos. Part B Eng..

[5] Eskenati A.R., Mahboob A., Alirezaie A., Askari R., Kolbadi S.M.S. (2021). Investigating the effect of longitudinal gallery on dynamical response of gravity concrete dams using fem. J. Southwest Jiaotong Univ..

[6] Al-Lami K., D’Antino T., Colombi P. (2020). Durability of Fabric-Reinforced Cementitious Matrix (FRCM) Composites: A Review. Appl. Sci..

[7] Wei L.-L., Zhu J.-H., Ueda T., Su M.-N., Liu J., Liu W., Tang L.-P., Xing F. (2020). Tensile behaviour of carbon fabric reinforced cementitious matrix composites as both strengthening and anode materials. Compos. Struct..

[8] Vedernikov A., Safonov A., Tucci F., Carlone P., Akhatov I. (2020). Pultruded materials and structures: A review. J. Compos. Mater..

[9] Zou X., Lin H., Feng P., Bao Y., Wang J. (2021). A review on FRP-concrete hybrid sections for bridge applications. Compos. Struct..

[10] Zou X., Feng P., Wang J. (2018). Bolted Shear Connection of FRP-Concrete Hybrid Beams. J. Compos. Constr..

[11] Nguyen H., Mutsuyoshi H., Zatar W. (2014). Push-out tests for shear connections between UHPFRC slabs and FRP girder. Compos. Struct..

[12] Correia J.R., Branco F.A., Ferreira J.G. (2009). Flexural behaviour of multi-span GFRP-concrete hybrid beams. Eng. Struct..

[13] Koaik A., Bel S., Jurkiewiez B. (2017). Experimental tests and analytical model of concrete-GFRP hybrid beams under flexure. Compos. Struct..

[14] Van Wingerde A.M., van Delft D.R.V., Knudsen E.S. (2003). Fatigue behaviour of bolted connections in pultruded FRP profiles. Plast. Rubber Compos..

[15] Mahboob A., Gil L., Bernat-Maso E., Eskenati A.R. (2021). Flexible Fiber Fabric for FRP–Concrete Connection of Thin Hybrid Slabs. Polymers.

[16] Mahboob A., Gil L., Bernat-Maso E., Eskenati A.R. (2021). Experimental and Numerical Study of Shear Interface Response of Hybrid Thin CFRP–Concrete Slabs. Materials.

[17] Khodaii A., Fallah S., Nejad Moghadas F. (2009). Geotextiles and Geomembranes Effects of geosynthetics on reduction of reflection cracking in asphalt overlays. Geotext. Geomembr..

[18] Meng W., Khayat K.H. (2016). Experimental and Numerical Studies on Flexural Behavior of Ultrahigh-Performance Concrete Panels Reinforced with Embedded Glass Fiber-Reinforced Polymer Grids. Transp. Res. Rec. J. Transp. Res. Board.

[19] Falliano D., De Domenico D., Ricciardi G., Gugliandolo E. (2019). Improving the flexural capacity of extrudable foamed concrete with glass-fiber bi-directional grid reinforcement: An experimental study. Compos. Struct..

[20] Corradi M., Borri A., Castori G., Sisti R. (2014). Shear strengthening of wall panels through jacketing with cement mortar reinforced by GFRP grids. Compos. Part B Eng..

[21] Dalalbashi A., Ghiassi B., Oliveira D.V., Freitas A. (2018). Fiber-to-mortar bond behavior in TRM composites: Effect of embedded length and fiber configuration. Compos. Part B Eng..

[22] Mahboob A., Eskenati A.R., Moradalizadeh S. (2021). Numerical Investigation and Cost Analysis of FRP-Concrete Unidirectional Hybrid Slabs. Int. J. Appl. Mech. Eng..

[23] Neagoe C.A. (2016). Structural Performance of FRP-Concrete Hybrid Beams with Flexible Shear Connection. Ph.D. Thesis.

[24] Mercedes L., Bernat-Maso E., Gil L. (2020). In-plane cyclic loading of masonry walls strengthened by vegetal-fabric-reinforced cementitious matrix (FRCM) composites. Eng. Struct..

[25] (2005). *EN 1504-3*. Products and Systems for the Protection and Repair of Concrete Structures—Definitions, Requirements, Quality Control and Evaluation of Conformity—Part 3: Structural and Non-Structural Repair. https://standards.iteh.ai/catalog/standards/cen/62c5607f-c564-4802-9b11-a872dc3c4b3c/en-1504-3-2005.

[26] Kent D.C., Park R. (1971). Flexural Members with Confined Concrete. J. Struct. Div..

[27] Di J., Cao L., Han J. (2020). Experimental study on the shear behavior of GFRP-concrete composite beam connections. Materials.

[28] Rajchel M., Kulpa M., Siwowski T. (2020). Experimental Study on a Novel Shear Connection System for FRP-Concrete Hybrid Bridge Girder. Materials.

[29] Gong J., Zou X., Xia P. (2019). Experimental Investigation of the Natural Bonding Strength between Stay-In-Place Form and Concrete in FRP-Concrete Decks/Beams. Appl. Sci..

